# Prediction and validation of nanowire proteins in *Oleidesulfovibrio alaskensis* G20 using machine learning and feature engineering

**DOI:** 10.1016/j.csbj.2025.04.022

**Published:** 2025-04-19

**Authors:** Dheeraj Raya, Vincent Peta, Alain Bomgni, Shiva Aryal, Tuyen Duc Do, Kalimuthu Jawaharraj, David R. Salem, Venkataramana Gadhamshetty, Saurabh Sudha Dhiman, Etienne Z. Gnimpieba

**Affiliations:** a Civil and Environmental Engineering, South Dakota Mines, Rapid City, SD 57701, USA; b 2Dimensional Materials for Biofilm Engineering, Science and Technology Center, South Dakota Mines, Rapid City, SD 57701, USA; c Data Driven Material Discovery Center for Bioengineering Innovation, South Dakota Mines, Rapid City, SD 57701, USA; d Biomedical Engineering Department, University of South Dakota, Sioux Falls, SD 57107, USA; e Chemical and Biological Engineering, South Dakota Mines, Rapid City, SD 57701, USA; f Chemistry Biology and Health Sciences, South Dakota Mines, Rapid City, SD 57701, USA

**Keywords:** Bacterial nanowires, Biofilm, Biologically informed neural network, Gene ontology, Machine learning, Protein sequence-based prediction, Random forest

## Abstract

The type-IV bacterial pili and multiheme c-type cytochrome protein family have gained significant attention due to their role in extracellular electron transfer (EET), which defines their electrogenic properties. These electrogenic behaviors play a crucial role in interspecies microbial communication, essential for microbial biofilm formation and the development of robust technologies such as biosensors. Given the technological and ecological significance of electron transfer mechanisms, this study presents NanowireML (NWML), a 2-staged machine learning (ML) system to identify and analyze nanowire (NW) proteins. Stage 1 predicts NW proteins using minimal features, and Stage 2 leverages graphical knowledge representation to predict the most relevant NW mechanism governing candidates in specific experimental conditions. To train the stage 1 model, we used a comprehensive dataset of 999 proteins from a public database specializing in NW development. The primary objective of the NWML model is to identify and validate microbial proteins involved in the biogenesis of NW. Protein feature, such as dipeptide amino acid composition, transition, and distribution enhance the model’s performance. Gene ontology (GO) analysis revealed that NW are structural extrusions, part of membranal proteins, with several exposed metal ion binding motifs. The predicted NW protein collection advances to stage 2, where their GO knowledge is stored using a graphical representation. A customized deep neural network (biologically influenced neural networks: BINN) then predicts the most relevant biological pathways governing NW formation using experimental gene expression data. Our study provides detailed insights into gene sets, unveiling the mechanistic networks and pathways crucial for NW formation. These findings enable data-driven decisions for biomedical and biotechnological applications. The NWML model demonstrated high accuracy achieving 94.87 %, 96.68 %, 96.65 %, 96.05 %, and 96.13 % on support vector machine (SVM), random forest (RF), extreme gradient boosting (XGBoost), logistic regression (LR), and artificial neural network (ANN).

## Graphical Abstract

**Figure:**
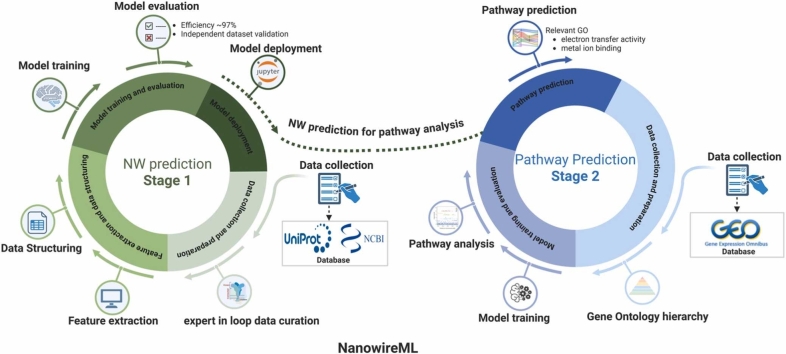

## 1 Introduction

An extracellular electron transfer (EET) is a process in which microbes transfer electrons within their microcosmic environments, including metals, which can occur through structural extensions known as nanowires (NW) or indirectly via electron shuttles [1]. Microbial NW, such as type-IV pili, are particularly intriguing due to their conductive properties, enabling microbes to transfer electrons over distances of up to 10 µm [2]. Owing to their electrogenic characteristics, microbial NW is essential for maintaining the microbial cell density, which subsequently regulates the biogeochemical cycling of the carbon, minerals, and interspecies energy exchange [3]. Moreover, the NW offers enormous potential for diverse biomedical and environmental applications, including wearable sensing devices, ultra-sensitive biosensors for biohazards, and next-generation conducting devices [4], [5], [6].

Several bacterial strains have been studied for their NW biogenesis and EET characteristics, including *Desulfovirbio desulfuricans* [7], *Desulfovibrio ferrophilus* [8], *Synechocystis* strain PCC 6803 [9], and *Pelotomaculum thermopropionicum* [9]. However, the mechanisms underlying NW formation have been thoroughly studied only in *Shewanella oneidensis* [10], [11] and *Geobacter sulfurreducens* [12]. Reports indicate that the PilA protein is responsible for the EET characteristics of type-IV pili, commonly referred to as NW or e-pili. Similarly, multiheme c-type cytochrome proteins, such as OmcS and OmcZ in *G. sulfurreducens* and MtrABC in *S. oneidensis,* play a crucial role in EET [13]. Notably, there are no existing reports validating the EET characteristics of *Oleidesulfovibrio alaskensis* G20 (formerly *Desulfovibrio alaskensis* G20*,* OAG-20), a prominent corrosion causing microbe [14] responsible for microbially induced corrosion (MIC), which costs the U.S. economy approximately $2 billion annually.

The structural and functional complexity of NW demands enormous biochemical validation datasets to elucidate EET characteristic in OA-G20. However, recent advances in data science allow leveraging machine learning (ML) models to rapidly characterize biomolecules, including proteins. ML a branch of artificial intelligence (AI), systematically applies algorithms such as random forest (RF) [15], support vector machine (SVM) [16], extreme gradient boosting (XGBoost) [17], logistic regression (LR) [18], multilayer perceptron (MLP); feed-forward artificial neural network (ANNs) [19], and more, deciphering the underlying correlation between existing datasets and uncharacterized properties of targeted molecules. Thus, leveraging existing genomics and proteomics datasets to deduce complex biological information has firmly established ML as a transformative tool for predicting various protein domains, including their classifications as antibiofilm [20], [21], [22], [23], quorum sensing [24], [25], antifreeze molecules [26], microbial corrosion [27], and more. Recently, an SVM-based ML model trained on experimentally validated antibiofilm peptides achieved 97.83 % accuracy in predicting antibiofilm peptide sequences [28]. Another study used a dataset comprising 122 molecules to successfully train an ML algorithm capable of predicting biofilm inhibitory properties [29]. Despite the promising progress of ML models in all biological domains, no existing ML models can predict and classify NW-forming proteins. Tools such as FlaFind [30] and PilFind [31] can detect cleavage sites in pili proteins; the functionality of these models is limited. Furthermore, PilFind is designed to identify all type-IV pili proteins, even though only specific types form e-pili. As a result, PilFind cannot effectively screen for multiheme cytochrome-C NW proteins.

The classification and sequence analysis of proteins is a multistep process that relies on numerical features (e.g., amino acid composition) and labels (e.g., positive/true or negative/false). These features are obtained by transforming the protein and peptide sequence into mathematical expressions, capturing intrinsic correlation with structural and functional attributes [32]. Understanding how each feature contributes to an ML model improves experimental design and minimizes false positive results. Several features (descriptors) have been introduced to improve ML predictive capabilities, such as amino acid composition (AAC), dipeptide composition (DPC), tripeptide composition (TPC), and more. The autocorrelation features, e.g., Moran and Geary, have also been implemented for sequence-based protein classification [21], [22], [24], [26], [33], [34], [35], [36]. Hence, protein-based sequence features and an ML approach hold potential for predicting protein properties. Incorporating Gene Ontology (GO), a comprehensive database of gene functions, further strengthens ML models by enabling the prediction of protein functionalities [37].

While ML models are widely used across various applications, a significant drawback is their 'black box' nature, which makes their internal mechanisms difficult to interpret. Deep neural networks (DNNs) are often considered black-box models due to their complex, multi-layered architectures, whereas ensemble decision tree models like random forest offer some degree of interpretability. For an in-depth understanding of NW biogenesis, examining the genes, biological processes, and molecular functions is imperative. This comprehensive investigation will elucidate the individual gene set involved and unveil the dynamic networks and pathways crucial for forming NW. The integration of ML in proteomics and transcriptomics workflows, especially using biologically informed deep learning models, has been pivotal in identifying key biomarkers and biological pathways. This approach enables the systematic integration of proteins, genes, and biological pathways into ML frameworks. For instance, graph neural networks (GNNs) have been successfully applied in biomedical research, such as in COVID-19 and prostate cancer studies. These GNN approaches have been implemented in frameworks such as biologically informed neural networks (BINN) and biologically influenced deep learning models (P-NET) [38], [39]. Moreover, these integrated models with pathway hierarchy enhance the comprehensibility of decision-making, a feature often lacking in neural networks, rendering them more interpretable than their neural counterparts. To enhance interpretability, BINN and P-NET establish connections between their layers and biological processes, represented in the form of graphs [38]. This approach renders the model's decision-making process interpretable, making the decision-making process more transparent.

With a long-term vision of creating an efficient ML-backed predicting protein functionality facility (PPFF), we have developed NWML, an automated two-stage ML framework to eliminate extensive biochemical validation. In stage 1, a probable and confirmed NW protein dataset was compiled from databases (DB). In stage 2, BINN was implemented using transcriptomics data and GO hierarchies to identify key pathways involved in NW formation. This comprehensive approach elucidates the individual gene set and unveils the biological pathways involved in the NW formation and its EET characteristics.

## 2 Material and methods

**Fig. 1:**
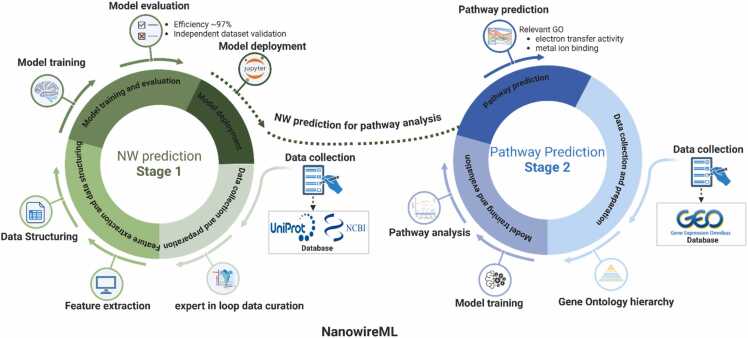
The complete workflow of the two-stage NWML framework.

The two-stage ML models were designed in coordination, allowing a streamlined workflow between datasets presenting potential NW proteins (Stage-1) and the prediction of the molecular mechanism involved in NW biogenesis (Stage-2) (Fig. 1). The designed sequential characteristic of the model was chosen for its broad-spectrum applications among users deciphering new biomarkers in microbial and cellular engineering. Stage one of the NWML model involved data preparation, feature extraction, and model development and validation, whereas stage two focused on implementing and optimizing the BINN.

### 2.1 Dataset preparation

Proteins involved in NW biogenesis were identified by manually curating amino acid (AA) sequences available in DBs such as UniProt [40] and NCBI [41] to create a dataset. A dataset is a systematically organized collection of biological sequences derived from established protein sequence DBs, assembled to support subsequent analysis and research. Initially, the “Nanowire” keyword was searched in targeted DBs, resulting in numerous redundant entries [40]. Rigorous manual curation was performed on the extracted AA sequence entries using open source, local self-hosted NLP tool docanno (https://github.com/doccano/doccano), resulting in the retention of 842 potential NW proteins. Similar searches and subsequent steps were conducted using targeted DBs, and 157 AA sequence entries were retained [41]. Each relevant sequence represents a single entry, and the compilation of all relevant entries constitutes one dataset. A sum of 999 proteins was retrieved and compiled to prepare a “Positive” dataset to enrich the ML model.

Similarly, the negative dataset was developed using the keyword “RecA” for various microbes from targeted DBs. RecA was chosen based on the need for a negative dataset that would not overlap with the positive dataset, which consists of proteins involved in NW formation. The RecA protein, which plays a key role in DNA repair and homologous recombination, is functionally unrelated to NW formation and, thus, serves as an appropriate representative for the negative dataset. A total of 999 protein entries were selected to ensure a balance between the positive and negative datasets for subsequent analysis. These entries were manually curated to ensure they did not overlap with the positive dataset. To maintain a balanced dataset, entries from DBs were randomly selected from targeted protein pools of RecA, ensuring that the number of negative and positive datasets is consistent. Both positive and negative datasets were used for the ML model's training and validation (testing). Approximately 70 % of the datasets (∼699) were used for training, and the remaining 30 % (∼300) was assigned as testing data.

### 2.2 Gene ontology analysis

The GO elucidates the interconnection among genes from different sources. The GO terms also represent correlations between the molecular functions of genes and their classified biological processes [42]. The positive dataset used for NWML model training was categorized based on specific GO terms, e.g., biological process (BP), molecular function (MF), and cellular component (CC) mined from the InterPro and UniProt DBs [40], [43]. Any NW sequence entry without annotated GO terms was not considered for GO analysis. The outcomes of the GO analysis are represented as a chord diagram (Fig. 3), highlighting the correlations between GO terms and the GO category. Python was used to analyze and generate predicted results for standardizing the NWML model using the Scikit learn package.

### 2.3 Protein features extraction

Twenty-one diverse features were extracted using iFeatureOmega [44]—a python package, to encompass a wide array of input characteristics, e.g., autocorrelation features, protein sequence features, and allied attributes of the NW proteins. The amino acid composition feature groups, mainly amino acid composition (AAC) [45], dipeptide composition type 1 and type 2 (DPC1 and DPC2) [46], and tripeptide composition type 1 and type 2(TPC1 and TPC2) [47] features, were extracted to represent the percentage of each amino acid in protein sequences. DPC1 computes the normalized frequency of all possible dipeptides (400 combinations) by dividing each dipeptide's count by the total number of dipeptides in the sequence, whereas DPC2 records the absolute count of each dipeptide without normalization. Similarly, TPC1 calculates the normalized frequency of all possible tripeptides (8000 combinations) by dividing each tripeptide's count by the total number of tripeptides in the sequence, while TPC2 records the absolute count of each tripeptide without normalization. These features provide insights into peptide occurrence patterns within protein sequences, capturing both relative and absolute representations of composition. Physicochemical properties of AA were extracted, describing parameters such as oxygen, sulfur, nitrogen, hydrogen, and carbon contents, as well as AA acidity and basicity, size, polarity, and charge percentage [48]. Autocorrelation features, including Moran, Geary, and NMBroto, were extracted, which describe the spatial autocorrelation of AA properties based on the amino acid indexes along the sequence [49]. Similarly, composition, transition, and distribution of AA sequence were extracted. In total, 19,857 features were extracted for each sequence of proteins reported in the positive and negative datasets. A detailed list of protein features covered in this study is summarized in Table 1.

**Table 1:** Comprehensive list of diverse features used for classification of nanowire-forming proteins.

Feature Category	Features in each category [Feature dimension]
Amino acid composition	Amino acid composition (AAC) [20]
	Composition of *k*-spaced amino acid pairs (CKSAAP) [1600]
	Dipeptide composition type 1 (DPC1) [400]
	Dipeptide composition type 2 (DPC2) [400]
	Tripeptide composition type 1 (TPC1) [8000]
	Tripeptide composition type 2 (TPC2) [8000]
Autocorrelation	Moran (Moran) [24]
	Geary (Geary) [24]
	Normalized Moreu-Broto (Moreau) [24]
	Cross variance [168]
	Auto covariance [24]
Quasi-sequence-order	Sequence-order-coupling number (SOCNumber) [6]
	Quasi-sequence-order descriptors (QSO) [46]
K-nearest neighbor	K-nearest neighbor(KNN) [20]
Aaindex	Aaindex [400]
Z-scale	Z-scale (Zscale) [400]
Pseudo-amino acid composition	Pseudo-amino acid composition (PAAC) [23]
Grouped amino acid composition	Grouped amino acid composition (GAAC) [5]
C/T/D	Composition (CTDC) [39]
	Transition (CTDT) [39]
	Distribution (CTDD) [195]

### 2.4 Features preprocessing and machine learning model

The feature extraction workflow yielded 19,857 features used to train the NWML model. To mitigate data leakage, several key techniques were implemented, including appropriate data splitting before feature scaling and the prevention of target leakage [50]. The algorithms used to develop the NWML model include SVM [16], RF [15], XGBoost [17], LR [18], and MLP [19]. Among all tested algorithms, the one that provided the best accuracy was considered the “Best Fit” and subsequently used for the NWML framework. Potential “overfitting” problems associated with the ML models were addressed using a five-fold cross-validation of the training (both positive and negative) datasets. The entire dataset was divided into five sub-sets for NWML training. Specifically, four sub-sets were used for training, and one sub-set was used for NWML testing. The testing includes performance accuracy, precision, recall, and F1 score—providing a comprehensive evaluation of its effectiveness in predicting NW proteins. These metrics are calculated based on true positives (correctly predicted NW proteins), true negatives (correctly identified non-NW proteins), false positives (incorrectly predicted NW proteins), and false negatives (missed NW proteins), ensuring a thorough validation of the model's predictive capabilities. Collectively, these metrics measure the (i) ability to identify all relevant instances (positive or negative), i.e., relevant AA sequences which could involve in the NW formation, (ii) accuracy of positive predictions, i.e., relative probability in forming the NW, (iii) overall correctness, i.e., ratio of validated proteins involved in NW formation over predicted proteins, and (iv) the test accuracy by integrating the precision and recall, providing a more comprehensive view of model’s performance. These indexes are expressed as:

(1) $$\mathrm{Accuracy}=\;\frac{\mathrm{TP}+\mathrm{TN}}{\mathrm{TP}+\mathrm{FP}+\mathrm{TN}+\mathrm{FN}}$$

(2) $$\mathrm{Precision}=\;\frac{\mathrm{TP}}{\mathrm{TP}+\mathrm{FP}}$$

(3) $$\mathrm{Recall}=\;\frac{\mathrm{TP}}{\mathrm{TP}+\mathrm{FN}}$$

(4) $$F1\mathrm{score}=2*\left(\frac{\mathrm{Precision}*\mathrm{Recall}}{\mathrm{Precision}+\mathrm{Recall}}\right)$$

Where TP (true positive) and TN (true negative) are correctly predicted positive (NW) and negative (non-NW) samples. FP and FN represent false positive and false negative entries representing the wrongly predicted NW and non-NW samples. The NWML model was evaluated by receiver operating characteristics and areas under the curve (AUC-ROC) (Fig. 4b) [51].

Considering the diverse nature of AA sequences that could function as NW proteins, a screening methodology was adopted using an independent dataset of NW protein details collected from the literature and analyzed with the developed NWML model (Table S1). The AA sequences in this independent dataset were not included in either the positive or negative datasets and thus are referred to as the independent dataset.

### 2.5 Biologically informed neural network for pathway analysis

In stage two (pathway prediction), the BINN [38] was employed to elucidate the underlying mechanisms that govern unified molecular pathways involved in NW biogenesis. BINN is a deep learning-based approach that integrates information from GO pathways, incorporating biological relationships (e.g., signal transduction) to enable a cohesive analysis of molecular functions (e.g., electron transfer activity) and biological processes (e.g., primary metabolic process).

To support this analysis, gene expression datasets from *G. sulfurreducens—*a model organism known for its NW biogenesis characteristics, were obtained from the NCBI Gene Expression Omnibus (GEO) database [52], [53]. The transcriptomic data included four NW-forming and four non-NW-forming phenotypes. The NW forming phenotype dataset (GEO accession number GSE200066) includes conditions (e.g., acetate, formate) for BINN analysis. These conditions produce NW, including electron donor with graphite poised at −0.07 V or −0.01 V compared to SHE (standard hydrogen electrode), or fumarate as the electron acceptor in biofilm and planktonic cells. Conversely, GEO accession number GSE179298 represents the non-NW-forming phenotype dataset for the model bacterium. BINN was broadly trained using the aforementioned GEO datasets and GO pathway data to validate the predictive capabilities of the NWML model. The NWML was analyzed using Shapley Additive Explanations (SHAP) [38], a feature attribution technique that calculates the Shapley values to quantify each node’s contribution to the prediction. In this context, each node corresponds to a gene or a biological or molecular pathway.

The differentially expressed genes (DEG) analysis was performed using the DESeq2 package [54], with acetate as an electron donor and fumarate as an electron acceptor in planktonic cells as the control for the NW-forming phenotype dataset. DEGs were functionally annotated with their corresponding proteins via the UniProt database [40]. Associated biological processes and molecular functions were retrieved from the GO repository to contextualize the DEGs within relevant pathways [42].

## 3 Results and discussion

### 3.1 NWML – An ML generalizable model for predicting NW-forming proteins

We developed NWML - a novel, generalizable ML model designed to predict microbial NW protein with an accuracy of up to 96.68 %. Additionally, NWML incorporates predictive pathway analysis on experimental data to help identify mechanisms of action behind NW formation.

In the first stage, the ML model was trained to predict proteins involved in the NW biogenesis. The training dataset included features such as physicochemical properties, sequence-based characteristics, and other relevant biological parameters. Several algorithms were evaluated, including SVM, RF, XGBoost, LR, and MLP. Each model’s performance was evaluated using standard metrics: accuracy, precision, recall, and F1 score. Among these, RF delivered the best performance and was selected as the core classifier for NWML. This stage effectively reduces the reliance on extensive biochemical experiments by offering a high-confidence method for identifying candidate NW proteins.

The second stage aimed at predicting the underlying mechanisms related to the formation of NW. Building on the predictions from Stage 1, stage 2 aimed to uncover the molecular pathways and biological interactions that drive these processes. Advanced ML techniques were employed to analyze complex, non-linear relationships within the data. The models were trained on datasets that included DEG and GO terms. By integrating experimental data with graph-based representations and deep learning, NWML offers a clearer understanding of the functional roles of predicted NW proteins, supporting targeted biomedical and environmental applications.

### 3.2 Analysis and characteristics of protein datasets

To develop the NWML model, we meticulously curated both positive (NW-forming proteins) and negative (non-NW-forming proteins) datasets. The selection of negative datasets often needs a standardized rule; typically, a common practice is ensuring that negative datasets do not overlap with positive ones.

For positive datasets, the manual curation process, which was informed by studies documenting the importance and functional relevance of these proteins, ensures the robustness of our dataset. Baquero et al. highlighted the widespread presence of extracellular cytochrome NW in microbial communities, reinforcing the relevance of our dataset. This evidence reinforces the relevance and accuracy of the dataset used in our study reflects biologically significant patterns observed in nature [55]. In positive datasets, manual curations were focused on including those proteins that were either from the type-IV pili or exhibited specific structural domains crucial for the functionality of the NW. The positive dataset comprises 32 entries containing PilA-type proteins, 911 entries with Nanowire_3heme (IPR026352) domains, and 819 entries featuring multihaem_cyt_sf (IPR036280) domains (Fig. S1). These domains are strongly implicated in the biogenesis of NW, structures that play a pivotal role in microbial EET characteristics. Another example includes Nanowire_3heme (Interpro domain: IPR026352), critical for binding with the surrounding three heme groups in *G. sulfureducens* (Gene ID: GSU_1996) [56]. Additionally, we included well-established NW-forming proteins such as OmcS, OmcZ, MtrA, OmcA, and PilA, all of which actively participate in NW biogenesis and EET in *G. sulfurreducens* [57], [58] and *S. oneidensis* [11]*.*

**Fig. 2:**
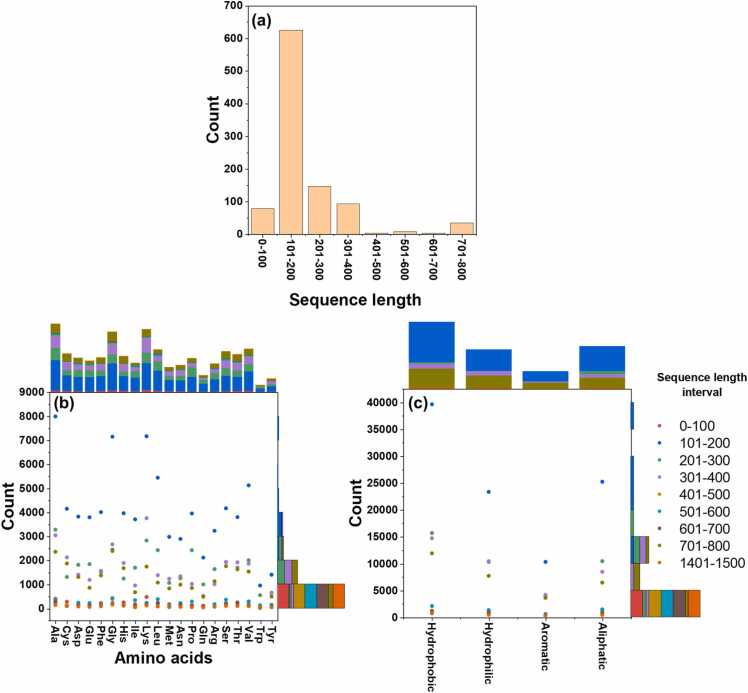
An overview of the sequence properties of nanowire-forming proteins extracted from UniProt and NCBI databases. The top inset (b,c) represents the total sum of each category shown on the x-axis; the side inset (b,c) represents the total sum of count within each interval in the y-axis. The representations include (a) sequence length distribution, (b) residue distribution within sequence intervals, and (c) residue properties classified as hydrophobic, hydrophilic, aromatic, and aliphatic.

Furthermore, the positive dataset comprised 852 sequences with relatively uniform lengths ranging from 50 to 350 amino acids (Fig. 2a). PilA protein, a constituent of type-IV pili, are typically ∼90 amino acids long [57], while multiheme proteins exhibit longer sequences exceeding 200 amino acids [58]. Remarkably, the OmcS and OmcZ cytochromes share no similarity in their structure or AA sequences [59]. Therefore, training and validating the NWML model is challenging due to dataset entries' complexity and variability based on multiple sequence alignment.

We further analyzed the AA composition of NW-forming proteins and observed significantly higher counts of alanine (A), lysine (K), and glycine (G), while tyrosine (Y), glutamine (Q), and tryptophan (W) were less abundant (Fig. 2b). These results align with previous studies demonstrating that PilA and OmcS derived NW contain positively charged residues, facilitating interactions with negatively charged Fe (III) oxides, a crucial factor in Fe (III) reduction [62], [63] (Fig. 1c). Additionally, we examined the physicochemical properties of the positive datasets (e.g., hydrophobic, hydrophilic) and found a higher prevalence of hydrophobic residues (Fig. 2c), consistent with a previous report highlighting the predominantly hydrophobic nature of cytochrome-based NW [60]. Since our primary goal was to predict and validate the NW proteins, the negative dataset was not analyzed further.

### 3.3 Functional roles of cytochrome and pili in nanowire

NWs exhibit unique EET traits over extended distances of up to 10 µm, essential for bioprocesses such as anaerobic respiration, biogeochemical cycling, and bioenergy production [61]. In-depth molecular and sequence analysis of NW-forming proteins will reveal the key AA sequence (considered as a “feature” for NWML) contributing to their EET properties. Furthermore, screening of GO terms effectively narrows NW proteins based on their specific roles. GO terms such as electron transport chain (ETC), cell outer membrane, and metal ion binding are primary indicators of NW functions. Therefore, the positive datasets were categorized based on GO terms, i.e., BP, CC, and MF, to identify critical AA sequences.

**Fig. 3:**
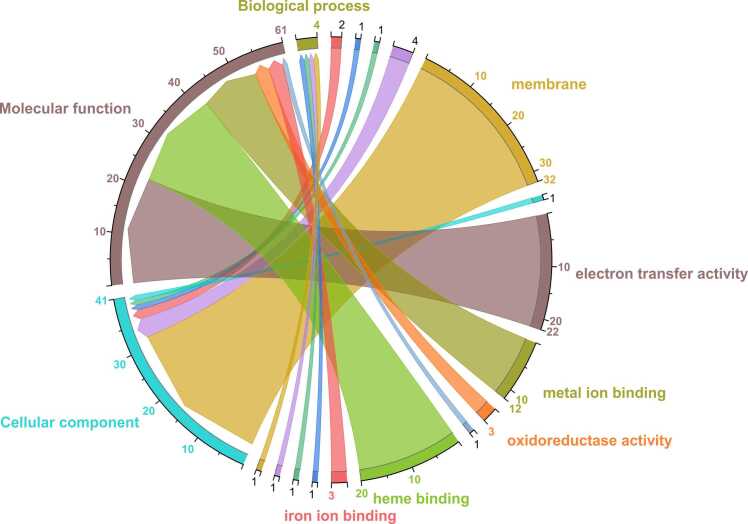
Chord diagram illustrating the associated gene ontology (GO) term of nanowire-forming protein dataset and their respective functional categories: biological process, molecular function, and cellular component. Each arc denotes a GO term, while the ribbon connecting the opposite end of the arc represents the functional category. The thickness of each ribbon corresponds to the frequency of the respective GO term.

The GO term BP represents protein-based pathways, MF describes biochemical activities, and CC identifies a protein’s subcellular localization [42]. However, due to limited data annotations, essential GO terms associated with each protein in the positive dataset were not readily available from UniProt [62]. Therefore, GO terms were retrieved from UniProt and evaluated using Python, which accounts for 3.43 % of all sequences in the positive dataset (Fig. 3).

Given that NW connects insoluble metal oxides with ETC, a key GO term related to BP, we examined the genes linked to the ETC and EET within the positive datasets. For instance, the expression of MtrC and OmcA protein-based NW in *S. oneidensis* showed a strong correlation with cellular reductase activity, which indicated their role in the ETC [13]. However, only four proteins, OmcC, PilA, Cytochrome c522, and PilA-N, within the positive dataset exhibited associated BP terms, including ETC, suggesting an enrichment of ETC associated NW proteins.

The GO term CC is essential in NW research, as it aids in pinpointing the specific subcellular locations where NW-associated proteins, such as PilA or cytochrome, are expressed. Because these proteins initiate the electron transfer mechanisms, membranal proteins, viz. outer membrane cytochrome proteins, OmcS, and PilA, are the rate-limiting components regulating the EET characteristics [11]. This localization is critical for understanding the functional roles of these proteins in electron transport processes, with the outer membrane serving as the initial site for NW biogenesis. Consistent with this, CC enrichment analysis confirmed a strong representation of membrane-associated proteins (Fig. 3), reinforcing their central role in controlling EET.

For GO term MF, an in-depth analysis of the three dominant terms was considered: (i) electron transfer activity (GO:0009055), (ii) metal ion binding (GO: 0046872), and (iii) heme binding (GO: 0020037). The GO term GO:0009055 (electron transfer activity) is a characteristic feature of a NW protein [9], [60].

The GO term GO: 0046872 (metal ion binding) is vital in maintaining the three-dimensional conformation of the NW, facilitating their interactions with insoluble metal oxides present in the surroundings [61], [63]. Proteins such as OmcS and PilA provide supporting evidence, as they contain positively charged domains that can bind with negatively charged metal oxides [64], [65]. These interactions between metal oxides and NW help facilitate the EET when an insoluble electron acceptor is in consideration [63]. The GO term metal ion binding refers to the molecular function of proteins capable of selectively binding metal ions [42]. This function is crucial for NW due to their frequent ability to bind with metal facilitating in EET. Heme within OmcS is essential for its functioning as an electron transfer conduit, as iron-containing heme acts as a redox-active center for accepting or donating electrons in NW [66]. These cytochromes act as oxidoreductive enzymes catalyzing the reduction of various reactive substrates formed from polypeptide (monomer) after CXXCH motifs within the polypeptide are covalently attached with heme [66]. In the case of OmcZ, heme six is prominently exposed to the solvent in each filament subunit. In contrast, within the OmcE and OmcS, only the hemes at the two ends of a filament are solvent exposed. The multiple exposed hemes of OmcZ could make very close contact with the electrode surface [59].

The GO term heme binding (GO:0020037) is crucial for NW polymerization. In the case of cytochrome-based NW, OmcS and OmcZ proteins are involved, which contain multiple heme groups. For example, each OmcS monomer harbors six heme-binding motifs, while OmcZ has eight [67], [68]. The linear arrangement of these heme groups is critical as each is sequentially linked to the next, enabling directional and efficient electron transfer along the cytochrome-based NW [69]. The structure of these NW reveals interconnected chains of cytochromes enclosing stacked heme cofactors aligned in parallel (3.4–4.1 Å) and T-stacked (5.4–6.1 Å) sequential pairs. Such closely packed heme pairs facilitate rapid conduction across extended distances [68]. The hydrogen bond network on cytochrome-based NW and heme planarity highly impacts the heme reduction potential, increasing the electron transfer rate in NW [70]. Given that NW formation occurs during the limitation of electrons, these structural properties facilitate at least two major roles of NW proteins in electron transfer activity and metal ion binding [60], [61].

### 3.4 Model performance with features subsets

To convert the protein sequence into numeric values compatible with the NWML model, we utilized the iFeatureOmega package for feature extraction [44]. iFeatureOmega is a versatile tool designed to extract various features from biological sequences, including proteins, DNA, and RNA, making it highly suitable for computational biology. The process begins with inputting biological sequences in a standard format, such as FASTA. iFeatureOmega extracts a comprehensive set of features encompassing sequence-based, physicochemical, and structural attributes [44]. A detailed list of all extracted features is presented in Table 1. We initially evaluated the classifier using each feature individually to assess its contribution and effectiveness in the classification process, as each feature has demonstrated real-world applicability across various biological domains. For instance, pseudo amino acid composition (PAAC) is extensively used in protein function prediction and subcellular localization analyses, providing a global overview of protein composition [71]. In addition, PAAC extends AAC by incorporating sequence-order information, thereby enhancing enzyme subfamily classification and biomarker discovery [72]. Global autocorrelation, such as Moran and Geary has been employed in remote sensing to analyze NDVI data and map spatial patterns of crop yield, thereby aiding yield prediction and soil management [73]. The AAC feature subset has demonstrated robust predictive capacity for identifying cancer cells, underscoring its potential utility in precision oncology [74]. This tailored feature subset study validated the functional importance of each group and ensured that our full feature set remains robust and informative for diverse applications in fields such as enzyme engineering, and structural bioinformatics.

Using the twenty-one extracted features, we built individual RF models with each feature serving as input (Figure S2). We selected the RF model as the starting point for developing the NWML model due to its robustness in handling high dimensional data and ability to provide reliable performance across diverse datasets [33]. The RF model works by constructing an ensemble of decision trees during training, where each tree is built on a random subset of features and data samples, and the final prediction is made by aggregating the outputs of all trees, typically through majority voting or averaging [15].

The accuracy observed for each feature exceeds the accuracy previously reported using RF models [26], [75], [76], suggesting that combining multiple features could further enhance prediction accuracy [26]. This high accuracy indicates that the model is proficient in recognizing and classifying the relevant patterns in the protein sequences based on the single features. Among the features, Moran, SOCNumber, Geary, and Auto covariance (AC) showed a comparatively lower accuracy of ∼94 % than the rest of the features. Moran and Geary are the measure of spatial autocorrelation, which assesses how similar residues are to each other based on their positions in the sequence [49]. A high correlation in spatial distribution suggests that similar residues are found close to each other within the NW protein sequence.

In contrast, the AAC feature group (AAC, CKSAAP, DPC, and TPC) achieved an accuracy of up to 99 %. These features include AAC, TPC, and DPC, which quantify the frequency of individual residues and pairs of residues in protein sequences. These features provide detailed information on the compositional features within the sequence, helping the model make more accurate predictions [77]. The AAC-based features have been widely applied in classifying protein subcellular localization [78].

The composition, transition, and distribution (CTD) feature group categorizes the AA into three classes for various physicochemical properties [79]. This comprehensive CTD framework comprises three key components: i) Composition (CTDC), which measures the overall proportion of residues from each class; (ii) Transition (CTDT), which captures the frequency of transitions between different classes along the sequence; and (iii) Distribution (CTDD), which describes how residues from each class are spatially arranged within the sequence. The CTDD descriptor group (CTDC, CTDT, and CTDD), Amino acid Index protein features, and Z-scale protein features showed higher accuracy up to ∼99 %. The CTDD group and amino acid index protein features have been widely used to predict protein functions [80]. Similar performance trends in precision, recall, and f1 score were observed for each input feature (Figure. S2). These results indicate that all features significantly contribute to the model’s performance in classifying NW proteins, highlighting their importance in constructing a predictive model.

### 3.5 Model development and evaluation

To develop the NWML model, structural and functional details of the protein’s structure (i.e., primary, secondary, etc.) were leveraged. The prime aim of ML models was to identify and understand the features (e.g., DPC, TPC) unique to NWs. A total of 19,857 features obtained from twenty-one protein features (Table 1) for each protein sequence in the dataset were used to train stage 1 of the NWML model (SVM, RF, LR, XGBoost, and MLP).

The SVM model was implemented with a radial basis function (RBF) kernel and no recursive feature elimination, while LR and MLP employed default parameters. The accuracy of the trained model on the dataset achieved an accuracy of 94.87 %, 96.68 %, 96.65 %, 96.05 %, and 96.13 % for SVM, RF, XGBoost, LR, and MLP, respectively, following five-fold cross-validation (Table 2). The consistency in performance across folds indicated that none of the models suffered from overfitting. Overall, RF achieved higher accuracy than other models, e.g., SVM, LR, XGBoost, and MLP and hence, was used in the first stage of the NWML framework. The AUC-ROC curves for all models are shown in Table 2. AUC-ROC, one of the popular techniques to measure the performance of ML models, provides insights into decision-making across different levels of certainty. This method utilizes a two-dimensional probability curve that plots the true positive rate (TPR) against the false positive rate (FPR) at various thresholds. While SVM is widely employed in peptide prediction, which excels in binary classification problems and is recognized for its robustness, it showed the lowest accuracy compared to all the tested models.

**Table 2:** Evaluation metrics for various classification models.

Classifier	Mean Accuracy	SD (Accuracy)	Precision	Recall	F1-score	ROC AUC
SVM	0.948701	0.008623	0.950758	0.922794	0.936567	0.969615
Random Forest	0.966858	0.007289	0.992063	0.919118	0.954198	0.982625
XGBoost	0.966503	0.00766	1	0.915441	0.955854	0.985652
Logistic Regression	0.960537	0.002501	0.976378	0.911765	0.942966	0.990255
MLP Classifier	0.961343	0.010041	0.984252	0.919118	0.95057	0.992025

Mean Accuracy: Average classification accuracy across multiple runs.

Standard deviation (SD) Accuracy: Standard deviation of accuracy, indicating variability.

Precision: Proportion of correctly predicted positive instances out of all predicted positives.

Recall: Ability of the model to identify all actual positive instances.

F1-score: Harmonic mean of precision and recall, balancing false positives and false negatives.

ROC AUC: Area under the Receiver Operating Characteristic curve, measuring overall classification performance.

On the other hand, RF leverages a collection of decision trees, with each tree learning classifications based on a random subset of features. The most discriminating feature for each tree is chosen from a randomly selected subset of m features, where m is significantly smaller than the total number of features at each splitting or decision node. This contributes to RF's high robustness in predicting complex models through its ensemble learning approach, wherein multiple decision trees are combined to reduce overfitting and capture diverse aspects of the data. The improved accuracy achieved in the RF model is due to its ability to capture complex non-linear relationships, making it suitable for classifying NW proteins. In contrast to the single input feature-based ML model, the performance of the ML model declined when using a combination of all features. As the dataset’s features increase, the data becomes sparser within the feature space, creating challenges in accurately estimating model parameters and raising the risk of overfitting. Training an ML model with many features often performs poorly, a phenomenon known as the “curse of dimensionality.” Figure S3 depicts the classification accuracy of the RF model for NW. A TPR at a low FPR reflects the robustness of the model in accurately identifying positive datasets while mitigating the misclassification of negative datasets. This scenario underscores the model’s sensitivity in detecting true positives while concurrently minimizing the occurrence of false detection. Such an outcome underscores the model’s efficiency in distinguishing between positive and negative datasets, exemplifying its capacity for accurate classification.

### 3.6 Performance check of developed ML models

**Fig. 4:**
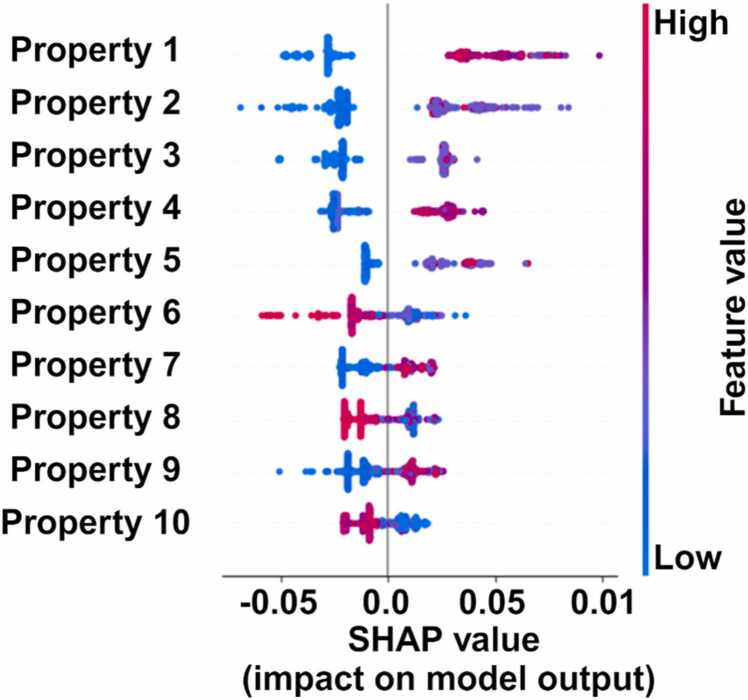
An overview of the top 10 selected features contributing to the decision-making process in the random forest model. Fig. 4. Shows Property 1: CKSAAP_CC.gap2; Frequency of occurrences of pairs of residues with a gap of 2 in the protein sequence, Property 2: DPC_CH.1; Frequency of occurrences of dipeptides formed by cysteine and histidine with a lag of 1 in protein sequence, Property 3: DPC_CH.2; Frequency of occurrences of dipeptides formed by cysteine and histidine with a lag of 1 in protein sequence, Property 4: AAC_C.1; Frequency of residue Cysteine in protein sequence, Property 5: DPC_CH.3; Frequency of occurrences of dipeptides formed by cysteine and histidine with a lag of 1 in protein sequence, Property 6: CC_BEGF750101_ANDN920101_lag.3; Cross-covariance between physicochemical properties BEGF750101 and ANDN920101 with a lag of 3 positions in the protein sequence, Property 7: CTDT_normwaalsvolume.Tr1331; Composition of transition based on normalized atomic van der Waals volume property, Property 8: CTDC_normwaalsvolume.G2; Composition of distribution based on the normalized atomic van der Waals volume property with a gap of 2, Property 9: GAAC_positivecharge; composition of residues with a positive charge in protein sequence.

Stage 1 of the NWML model exhibited strong prediction performance, underscoring the features’ effectiveness in characterizing NW (Fig. 4). To further interpret the model, permutation-based feature importance was assessed using the SHAP method (Fig. 4). In the SHAP summary plot, the horizontal axis represents SHAP values (i.e., the impact on model output), while the color gradient reflects the magnitude of feature values (blue for low, red for high). The vertical axis represents the hierarchical features based on their importance, with the most influential feature positioned at the top and the least influential at the bottom. Positive or negative SHAP values associated with features indicate their contribution type (positively or negatively) to classifying proteins forming NW. For example, CKSAAP, CC.gap2, DPC_CH.1, DPC_CH.2 and AAC_C.1 have low values on the left side, whereas higher values on the right side indicate increased values for these features contribute to NW classification (Fig. 4).

**Fig. 5:**
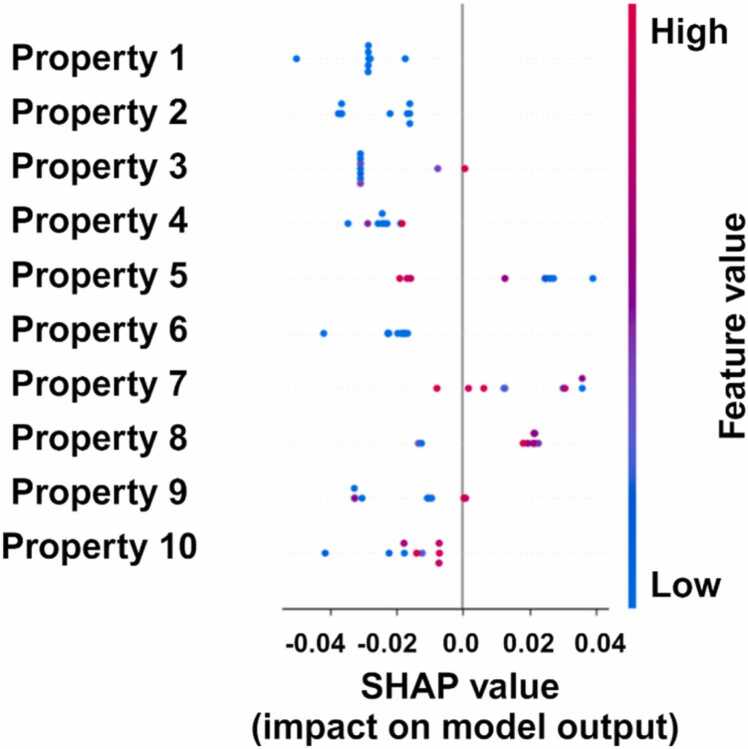
An overview of the top 10 selected features contributing to the decision-making process of the developed machine learning (random forest) model for an independent dataset. Fig. 5 shows Property 1: DPC_CH.2; Frequency of occurrences of dipeptides formed by cysteine and histidine with a lag of 1 in protein sequence, Property 2: CKSAAP_CC.gap2; Frequency of occurrences of pairs of residues with a gap of 2 in the protein sequence, Property 3: PAAC_Xc1.H; Pseudo amino acid composition feature related to group Xc1.H, Property 4: AAC_C.1; Frequency of residue Cysteine in protein sequence, Property 5: CC_BEGF750101_ANDN920101_lag.3; Cross-covariance between physicochemical properties BEGF750101 and ANDN920101 with a lag of 3 positions in the protein sequence, Property 6: DPC_CH.1; Frequency of occurrences of dipeptides formed by cysteine and histidine with a lag of 1 in protein sequence, Property 7: CTDD_hydrophobicity_ZIMJ680101_lag.3; Distribution based on the hydrophobicity property at position 50 in the protein sequence, Property 8: NMBroto_BEGF750102.lag2; Normalized Moreau-Broto autocorrelation for the property BEGF750102 with a lag of 2 positions in the protein sequence, Property 9: AAC_H; Frequency of the amino acid Histidine in the protein, Property 10: GAAC_positivecharge; composition of residues with a positive charges in protein sequence.

Among the top-performing features were three DPC descriptors, highlighting the role of dipeptides in distinguishing NW proteins. In addition, features from the CTD category were prominent, emphasizing their effectiveness in characterizing NWs. These findings underscore the key features that play a role in model performance, shedding light on the essential characteristics that define the NW-forming proteins (Fig. 4). Considering the growing class of NW protein [81], a validation of NW forming protein was performed using the independent NW forming protein details collected from the literature using the developed stage 1 of the NWML model (Table S1). The sequences in this independent dataset were absent in the positive or negative dataset, hence termed an independent dataset. The NWML model predicted nine out of ten proteins in an independent dataset belonging to the NW proteins (Fig. 5). Given the literature-verified nature of the dataset, biochemical characterization is not needed to predict results.

It is important to outline the limitations inherent in traditional methods, viz. BLAST and multiple sequence alignment tools when attempting to recognize the functionality of NW proteins. While these tools have proven invaluable in investigating the protein and nucleotide sequence details, their efficacy in NW classification may be compromised due to several factors, such as limited sequence or structural similarity among NW proteins. One crucial consideration is NW proteins’ intricate and multifaceted nature, which often possesses unique structural criteria and functional domains that conventional sequence-based alignment approaches may not fully capture. To ensure the validity of the independent dataset, step-by-step guidance is provided to utilize the NWML model, available on GitHub at https://github.com/bicbioeng/nanowire-protein-prediction. Further details are provided in ‘Data and Code Availability’ along with the manuscript.

### 3.7 Pathway analysis using BINN

Graph-based methods provide an effective way to visualize and interpret biologically complex data. These methods help identify links and interactions hidden in typical tabular data formats by visualizing genes and their molecular functions as nodes and edges in a network. In this study, transcriptomics data from *G. sulfurreducens* were analyzed to generate datasets representing NW forming conditions. *G. sulfurreducens*, a model organism extensively studied for its NW, typically produces NW during acetate as an electron donor and with a graphite electrode or fumarate as an electron acceptor [68], [82]. These datasets were combined with the GO pathway database to create and train BINN. The GO database provides information on the relationships between biological entities, including pathways and high-level processes [42]. To support a neural network-like structure, the graph was first subsetted and layered, then translated into a sparse neural network architecture. Each node was annotated with transcriptomics data, biological pathways, and molecular functions [38]. A network for NW forming conditions was generated with four hidden layers. The significance of each node can be understood by assessing how much the predictions deteriorate upon removing that specific node.

#### 3.7.1 Leveraging GO terms for pathway analysis

**Fig. 6:**
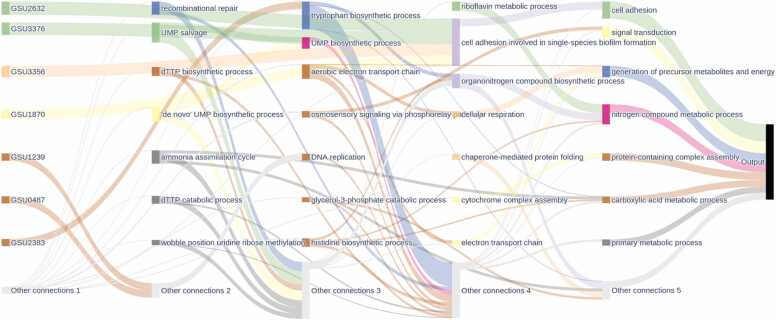
Sankey diagram illustrating key biological processes associated with nanowire formation. The diagram maps specific genes (left) to their corresponding gene ontology biological processes (middle) and their broader functional categories (right). The thickness of the connecting lines represents the strength of associations between genes and processes**.**

The aim for integrating the GEO of *G. sulfurreducens* was to utilize datasets that could elucidate the DEG under different conditions of NW formation. Our analysis uncovered a variety of enriched GO terms, providing insight into cellular processes (Fig. 6). Signal transduction pathways [GO:0007165] were notably represented among the enriched GO terms (Fig. 6). These details underscore the intricate nature of cellular signaling and response mechanisms to environmental cues [83]. Previous research has demonstrated the upregulation of cyclic diguanylate (c-di-GMP) during the formation of NW and biofilm formation by *G. sulfurreducens* when grown on graphite electrodes [83]. Furthermore, the most significant proteins identified, including GSU3376, GSU3356, and GSU2632, are implicated in the synthesis of c-di-GMP. Similarly, *S. oneidensis* MR-1 exhibited higher power density correlating with elevated levels of internal c-di-GMP [84].

Furthermore, the enrichment in GO terms related to cell adhesion [GO:0007155] highlighted the importance of NW in biofilm formation. Moreover, NW is well known for its multifaceted role in biofilm formation, cellular adhesion, and motility, and it facilitates the transport of a diverse array of substrates from DNA to effector proteins [82], [85]. The upregulation of c-di-GMP synthesis, supported by enriched GO terms and involvement of GSU3376, GSU3356, and GSU2632, suggests a key regulatory mechanism driving NW formation [86] (Fig. 6). Elevated c-di-GMP levels are crucial for regulating genes associated with cellular adhesion, biofilm formation, and extracellular electron transfer processes [86]. Additionally, the enrichment of genes associated with transmembrane transporter activity suggests that NW formation may involve the secretion of extracellular polymeric substances that contribute to biofilm matrix formation [86] (Fig. 6).

Carboxylic acid metabolism [GO:0019752] was also enriched, suggesting a role in maintaining energy balance (Fig. 6) [87]. This finding supports previous reports of an active tricarboxylic Acid Cycle (TCA) cycle in *G. sulfurreducens* under planktonic and biofilm conditions. The upregulated levels of TCA in *G. sulfurreducens* reveal the characteristics of producing energy and reducing potentials [87]. The resultant energy molecules and cofactors (i.e., NADH, NADPH, and others) could be an electron donor in oxidative phosphorylation [88]. The correlation between the upregulated TCA cycle and energy molecule generation underscores an intricate regulation of AA metabolism and its impact on cellular physiology. The sustained activity of the TCA cycle under planktonic and biofilm conditions provides the necessary reducing power and energy for cellular processes, including NW synthesis [87].

**Fig. 7:**
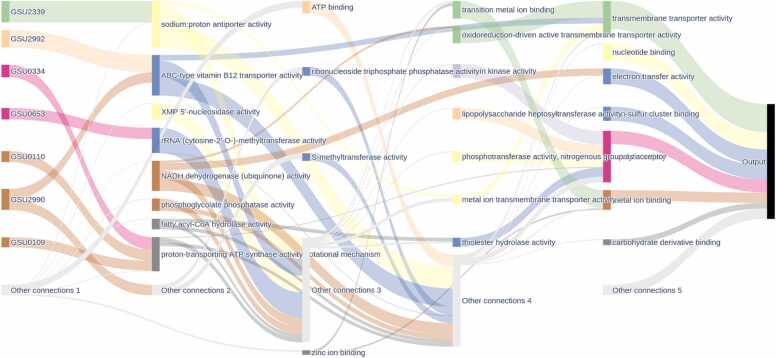
Sankey diagram illustrating key biological processes associated with nanowire formation. The diagram maps specific genes (left) to their corresponding gene ontology molecular function (middle) and their broader functional categories (right). The thickness of the connecting lines represents the strength of associations between genes and processes**.**

Additional molecular function analysis unveiled diverse enriched GO terms, each offering insight into cellular processes (Fig. 7). Metal ion binding [GO:0046872] emerged as a crucial aspect, indicating the ability of proteins to bind with metal ions vital for enzymatic and structural roles within the cell (Fig. 7) [61], [65]. Furthermore, there was a notable enrichment in transmembrane transporter activity [GO:0022857], underscoring the significance of molecular transport across cellular membranes (Fig. 7). pulGPQF genes encode proteins essential for the formation of the type II secretion system, which is necessary for reducing insoluble Fe(III) and Mn(IV) [89]. Notably, ABC transporters play pivotal roles in the export of molecules, e.g., EPS, across the periplasm, thereby contributing significantly to the formation of electroactive biofilms [86]. These biofilms exhibit a preference for expressing membrane-associated proteins [88]. Moreover, catalytic activity [GO:0003824] prominently emerged among the enriched GO terms (Fig. 7). These processes encompass acetate uptake, acetate activation, the citric acid cycle, conversion of pyruvate to acetyl-CoA, as well as anabolic pathways involving phosphoenolpyruvate synthase and anaplerotic reactions [90]. In addition, acetate uptake genes exhibit strong differential expression under various conditions, such as formate, hydrogen, and acetate, followed by the upregulation of genes involved in acetate activation and downstream processes [91].

#### 3.7.2 NW protein prediction and graph-based predictive modeling of their mechanism

To further test the NWML model, the dataset used in the training of stage 2, which included gene identifiers, was passed through stage 1 of the model to predict NW proteins. The predicted proteins were then used as the sole input for predicting the underlying molecular mechanisms in stage 2.

**Fig. 8:**
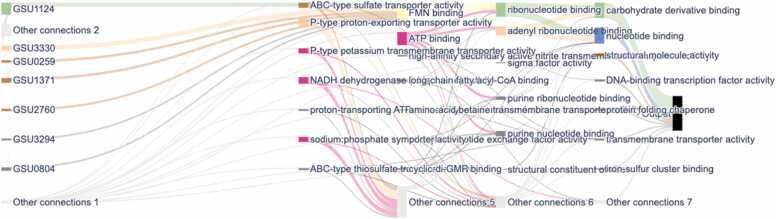
Sankey diagram illustrating key biological processes associated with nanowire formation. The diagram maps specific genes (left) to their corresponding GO molecular function (middle) and their broader functional categories (right). The thickness of the connecting lines represents the strength or frequency of associations between genes and processes**.**

Notably, transmembrane transporter activity [GO:0022857] was enriched, underscoring the key role of molecular transport across membranes (Fig. 8). These findings highlight the integral role of NW proteins in maintaining and facilitating essential biological processes. Furthermore, the model predicted various gene functions, such as iron-sulfur binding, ribonucleotide binding, and other key activities. The results demonstrate that the NWML model predicts both NW proteins and their predicted activities. This capability is crucial for understanding the complex network of NW protein interactions and their contributions to cellular processes. The prediction of these functions indicates that the model is a robust tool for identifying and elucidating the functions and mechanisms of NW proteins.

By using this model, researchers can anticipate how NW proteins function across diverse biological contexts. It enables in-depth predictions of NW protein activities and roles in specific cellular contexts. This predictive power can greatly improve the understanding of NW protein function and regulation, offering essential insights for fundamental research as well as practical applications in biomedical sciences.

## 4 Conclusion

Nontoxic, biodegradable NWs garner significant commercial interest as alternatives to synthetic nanoscale wires and carbon nanotubes. However, a thorough understanding of gene functions and the associated biological processes is essential to elucidate the mechanisms underlying NW biogenesis. Consequently, a rapid yet effective computational screening tool for validating the structural and functional characteristics of NW-forming proteins is both timely and transformative. The initial phase of the NWML setup provides an extensive collection of NW-forming proteins meticulously curated from existing literature and databases. GO analysis has demonstrated that NWs consist of membranal proteins with metal ion-binding domains crucial for electron transfer. Transcriptomics-driven metabolic pathway analyses confirm the pivotal role of c-di-GMP signaling in NW biogenesis. The trained Stage 1 of NWML achieved an impressive prediction accuracy of 96.68 %. This accuracy surpasses benchmarks typically reported with conventional alignment tools, which are considered reliable protein classification methods. Stage 2 of the NWML model aims to uncover latent insights and predictive indicators associated with significant pathways involved during NW protein synthesis by implementing advanced algorithms and computational frameworks in the subsequent phase. Through ML and data-driven methodologies, future research can explore new directions and deepen our understanding of complex biological processes such as NW biogenesis.

## CRediT authorship contribution statement

**Dhiman Saurabh Sudha:** Writing – review & editing, Supervision, Resources, Methodology, Funding acquisition, Conceptualization. **Gnimpieba Z. Etienne:** Project administration, Methodology, Investigation, Funding acquisition, Formal analysis, Conceptualization. **Raya Dheeraj:** Writing – original draft, Visualization, Validation, Methodology, Investigation, Data curation. **Duc Tuyen:** Visualization, Validation, Methodology. **Jawaharraj Kalimuthu:** Visualization. **Salem David R.:** Visualization. **Gadhamshetty Venkataramana:** Visualization. **Peta Vincent:** Visualization, Validation, Methodology. **Bomgni Alain:** Writing – original draft, Validation, Data curation. **Aryal Shiva:** Visualization, Validation, Methodology.

## Data and code availability of NWML model

The input dataset for this project relevant to the reproducibility of this paper is available at doi: 10.17632/vdvdrj3k2p.1.

The NW prediction code is available on GitHub at: https://github.com/bicbioeng/nanowire-protein-prediction; and the tool is accessible through the web server at: https://nanowire.bicbioeng.org/

## Funding

The authors acknowledge the financial support provided by the National Science Foundation (NSF) Award # 1849206 (2DBEST) and # 1920954 (DDMD). SD acknowledges the financial support from the SD EPSCoR Competitive Research Grant (2022) and Mining-Hub Dakota Gold industrial collaboration. Research reported in this publication was supported by SD BRIN and an Institutional Development Award (IDeA) from the National Institute of General Medical Sciences of the National Institutes of Health under grant number P20GM103443.

## Declaration of Competing Interest

There is no commercial or financial conflict to declare.
