# Supplementary material for: Prediction and validation of nanowire proteins in Oleidesulfovibrio alaskensis G20 using machine learning and feature engineering
Source: Comput Struct Biotechnol J. 2025 Apr 19;27:1706–18. doi: 10.1016/j.csbj.2025.04.022 (PMC12088768; doi:10.1016/j.csbj.2025.04.022)
Supplement: Supplementary file 1 — Supplementary material [file mmc1.docx]

Prediction and validation of nanowire proteins in *Oleidesulfovibrio alaskensis* G20

using machine learning and feature engineering model.

Dheeraj Raya,^1,2,3^ Vincent Peta,^3,4^ Alain Bomgni,^4^ Shiva Aryal,^4^ Tuyen Duc Do,^4^

Kalimuthu Jawaharraj,^1^ David R. Salem,^5^ Venkataramana Gadhamshetty, ^1,2,3^

Saurabh Sudha Dhiman,^1,2,3,6^* Etienne Z. Gnimpieba^2,3,4^*

^1^Civil and Environmental Engineering, South Dakota Mines, Rapid City, SD 57701, USA

^2^2Dimensional Materials for Biofilm Engineering, Science and Technology Center, South Dakota Mines, Rapid City, SD 57701, USA

^3^Data Driven Material Discovery Center for Bioengineering Innovation, South Dakota Mines, Rapid City, SD 57701, USA

^4^Biomedical Engineering Department, University of South Dakota, Sioux Falls, SD 57107, USA

^5^Chemical and Biological Engineering, South Dakota Mines, Rapid City, SD 57701, USA

^6^Chemistry Biology and Health Sciences, South Dakota Mines, Rapid City, SD 57701, USA

***Correspondence:**

Etienne Z. Gnimpieba

[Etienne.gnimpieba@usd.edu](mailto:Etienne.gnimpieba@usd.edu)

Saurabh Sudha Dhiman

[Saurabh.dhiman@sdsmt.edu](mailto:Saurabh.dhiman@sdsmt.edu)

**Supplementary Information:**

**Figure S1:** Distribution of domain counts in positive dataset

**Figure S2:** Accuracy, precision, recall, f1 score and roc curve of different protein features used for training the Random Forest machine learning model

**Fig. S3:** Confusion matrix for RF model

**
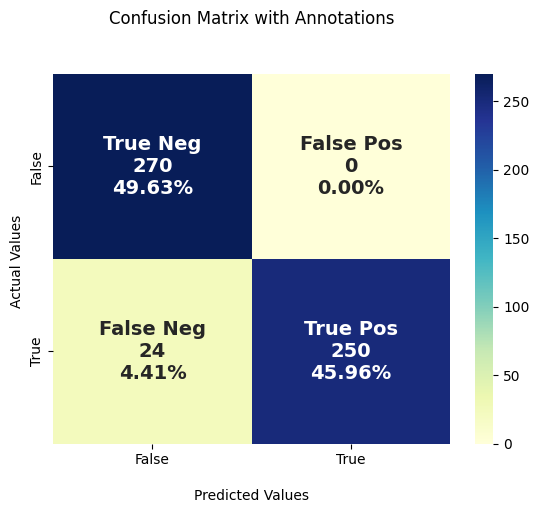
**

**Table S1:** List of protein along with the Gene ID used for the independent dataset

| Species | Gene ID | Reference |
| --- | --- | --- |
| [*Desulfovibrio ferrophilus*](https://www.uniprot.org/taxonomy/241368) | DFE_1797 | [1] |
| [*Syntrophus aciditrophicus (strain SB)*](https://www.uniprot.org/taxonomy/56780) | SYN_00814 | [2,3] |
| [*Desulfurivibrio alkaliphilus AHT2*](https://www.uniprot.org/taxonomy/589865) | DaAHT2_2283 | [2] |
| [*Geobacter metallireducens*](https://www.uniprot.org/taxonomy/269799) | pilA-N | [1] |
| *Calditerrivibrio nitroreducens* | CALNI_RS11510 | [2] |
| [*Geobacter uraniireducens*](https://www.uniprot.org/taxonomy/351605) | Gura_2677 | [1] |
| [*Flexistipes sinusarabici*](https://www.uniprot.org/taxonomy/717231) | Flexsi_2291 | [2] |
| [*Pelobacter propionicus*](https://www.uniprot.org/taxonomy/338966) | Ppro_1656 | [2] |
| *Desulfuromonas thiophila* | BLR80_RS13355 | [2] |
| *Geobacter soli* | SE37_07695 | [2] |

**References**

[1] Li Z, Chang W, Cui T, Xu D, Zhang D, Lou Y, et al. Adaptive bidirectional extracellular electron transfer during accelerated microbiologically influenced corrosion of stainless steel. Commun Mater 2021;2:67. https://doi.org/10.1038/s43246-021-00173-8.

[2] Walker DJ, Adhikari RY, Holmes DE, Ward JE, Woodard TL, Nevin KP, et al. Electrically conductive pili from pilin genes of phylogenetically diverse microorganisms. ISME J 2018;12:48–58. https://doi.org/10.1038/ismej.2017.141.

[3] Walker DJ, Nevin KP, Holmes DE, Rotaru A-E, Ward JE, Woodard TL, et al. Syntrophus conductive pili demonstrate that common hydrogen-donating syntrophs can have a direct electron transfer option. ISME J 2020;14:837–46.
